# Case report: Reversible Fanconi syndrome due to vitamin D deficiency in a patient with epilepsy harbouring a pathogenic variant in the SLC34A1 gene

**DOI:** 10.3389/fendo.2025.1553032

**Published:** 2025-03-28

**Authors:** Nicola Improda, Francesco Maria Rosanio, Luigi Annicchiarico Petruzzelli, Gyusy Ambrosio, Gabriele Malgieri, Claudia Mandato, Maria Rosaria Licenziati

**Affiliations:** ^1^ Neuro-Endocrine Diseases and Obesity Unit, Department of Neurosciences, Santobono-Pausilipon Children’s Hospital, Naples, Italy; ^2^ Department of Emergency, Santobono-Pausilipon Children’s Hospital, Naples, Italy; ^3^ Pediatric Nephrology Unit, Department of Emergency, Santobono-Pausilipon Children’s Hospital, Naples, Italy; ^4^ Department of Medicine, Surgery and Dentistry “Scuola Medica Salernitana”, University of Salerno, Baronissi, Italy

**Keywords:** hypophosphatemic rickets, vitamin D deficiency, renal Fanconi syndrome, nutritional rickets, phosphate

## Abstract

We report on a 3-year and 5-month-old boy who was referred for suspected rickets, due to knee valgus deformity developed over the previous year. The child had a history of epilepsy well-controlled with phenobarbital. His psychomotor development and growth metrics were appropriate for his age. On admission, laboratory work-up revealed elevated alkaline phosphatase (1289 U/L) and parathyroid hormone (PTH) (417 pg/ml), normal corrected calcium (9,3 mg/dl) and creatinine (0,21 mg/dl), low phosphate (3,2 mg/dl), 25-hydroxy vitamin D (6 ng/ml) and 1-25 hydroxy vitamin D (13.4 pg/mL, nv 20-80) concentrations. Urinalysis indicated low tubular reabsorption of phosphate (TRP % 10,7), along with bicarbonate, uric acid and amino acid loss, consistent with renal Fanconi syndrome. Based on these results, a genetic form of renal tubular dysfunction was suspected, and thus a clinical exome sequencing was requested. In the meanwhile, the child was commenced on Joulie solution (70 mg/kg/day of phosphate), calcitriol (0.03 mcg/kg/die), and ergocalciferol (1000 IU daily). FGF-23 concentrations were found to be within the normal range, thus ruling out FGF23-dependent forms of rickets. Surprisingly, we observed a dramatic improvement in laboratory parameters within two weeks from the treatment initiation, including normalisation of phosphate and PTH concentrations and resolution of Fanconi syndrome, prompting discontinuation of phosphate supplements. Molecular analysis identified a *de novo* monoallelic mutation (C.1006 + 1 G>A) in the solute carrier family 34 member 1(SLC34A1) gene encoding a protein involved in actively transporting phosphate into cells via Na+ cotransport in the renal brush border membrane. However, even without phosphate substitution no further drops in serum phosphate concentrations and persistently normal proximal renal tubular function were observed. Moreover the rickets changes had almost healed six months after starting vitamin D supplementation. This case provides further evidence that vitamin D deficiency may rarely cause renal Fanconi syndrome, reversible upon vitamin D replacement. This is particularly relevant in children with risk factors for vitamin D deficiency, including use of anticonvulsants.

## Introduction

Renal Fanconi syndrome, also known as the Debré, DeToni, Fanconi syndrome is characterised by a global dysfunction of the proximal renal tubule, causing renal losses due to impaired tubular reabsorption of glucose, phosphate (PO4), amino acids, bicarbonates and other electrolytes (1, 2). In infancy and childhood, renal Fanconi syndrome is mainly caused by inborn errors of metabolism (especially cystinosis), where tubular involvement is invariably irreversible (1, 2). On the other hand, reversible causes are very rare in childhood and include drug toxicity, infections, chemical intoxication and severe malnutrition (1). It is well known that nutritional rickets caused by severe vitamin D deficiency may be associated with hypophosphatemia due to reactive hyperparathyroidism (3–5). However, in anecdotal cases, vitamin D deficiency has been reported to cause a global dysfunction of the proximal renal tubule, eventually leading to florid rickets (3–5). The mechanisms behind this rare condition are still far from clear. Here we report for the first time a case of reversible renal Fanconi syndrome secondary to vitamin D deficiency in a 3-year-old epileptic boy taking enzyme-inducing anticonvulsant therapy, aiming at raising awareness regarding the possible occurrence of such a severe condition in this category of patients, thus potentially improving the management and outcomes of similar scenarios. This report also provides a brief review of the existing literature regarding vitamin D deficiency-induced Fanconi syndrome, with a focus on pathogenic hypotheses.

## Case description

A 3-year and 5-month-old Caucasian boy was referred for suspected rickets due to a valgus deformity of the knees that had developed over the preceding year. His family history was negative for bone diseases or electrolyte disturbances. During infancy, he had no significant health issues and received regular oral vitamin D supplementation (400 IU/day) from birth. At the age of 2 years, he was diagnosed with generalised epilepsy and was treated with phenobarbital (15 mg/day), achieving good seizure control. The patient’s dietary habits were normal for his age, including dairy products, and he spent time outdoors regularly for school and recreational activities. His growth was normal: weight 17 kg (-0.17 SDS), height 98 cm (0.91 SDS), BMI 17,7 kg/m² (1.46 SDS), and he had reached psychomotor milestones appropriate for his age. No systemic symptoms were reported.

**Figure 1:**
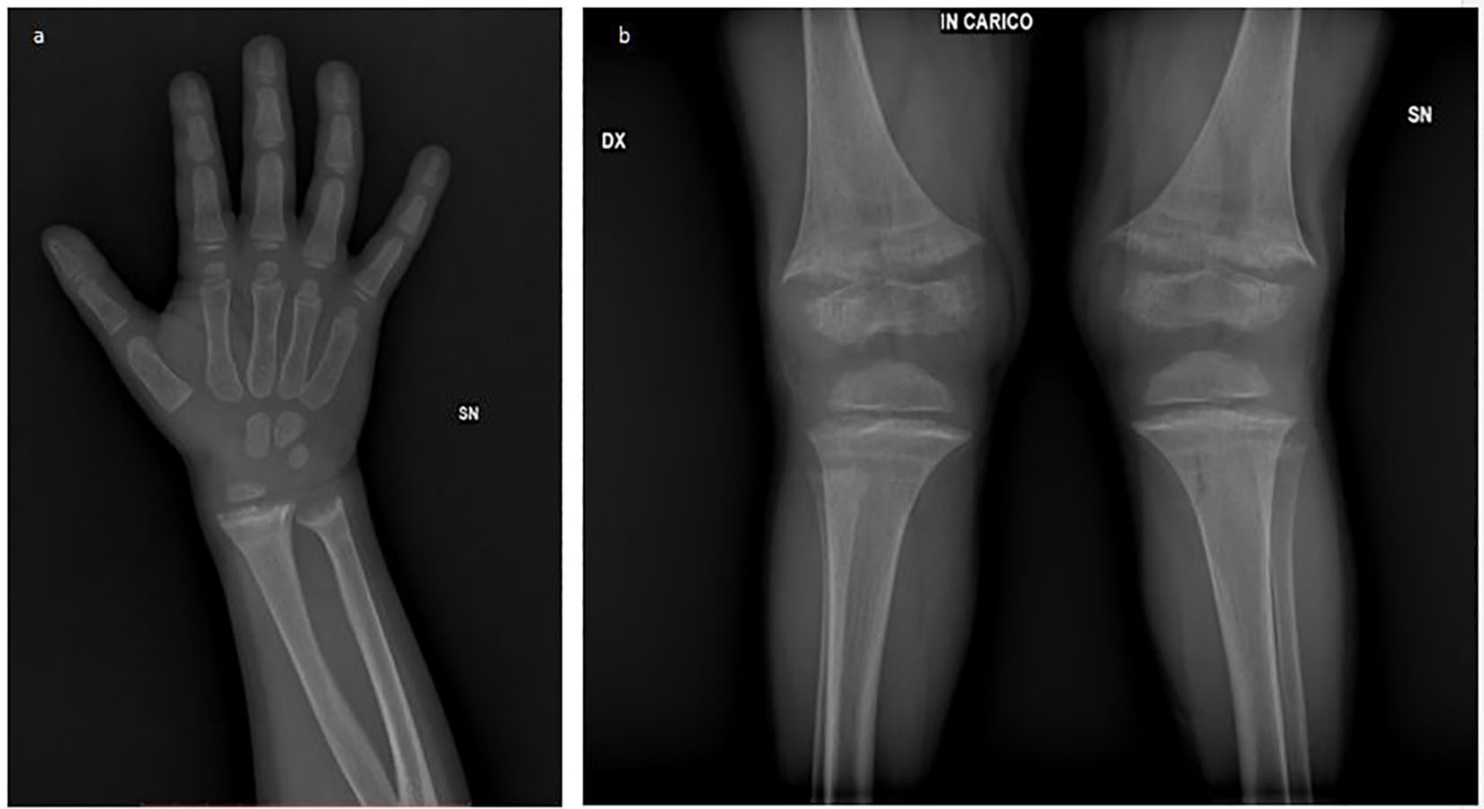
Radiographic features of rickets: **(a)** The rachitic wrist showing metaphyseal widening, irregular splaying, cortical thinning, and delayed ossification. **(b)** Bowing of long bones with genu valgus deformity and cup- or bell-shaped metaphyses.

X-ray imaging revealed classic rachitic changes in the wrist and knee (**Figures 1a, b**). An ultrasound did not show the presence of any morphological abnormalities of the kidney and urinary tract or nephrocalcinosis.

Laboratory tests showed: normal creatinine (0,21 mg/dl, nv 0,20-0,45), and corrected calcium (9,3 mg/dL, nv 8,6-11) concentrations, markedly low PO4 (3,2 mg/dL, nv 4,3-5,4), 25-hydroxyvitamin D, (25(OH)D) (6 ng/mL, nv 30.0 - 125.0), and 1,25-dihydroxyvitamin D (1,25(OH)_2_D) (13,4 pg/mL, nv 20-80) levels, along with elevated alkaline phosphatase (1289 U/L, nv 140-350), and parathyroid hormone (PTH) concentrations (417 pg/mL, nv 15.0 - 65.0) (**Table 1**). Urinalysis evidenced an alkaline pH (8), in the absence of glucose or proteins detectable.

**Table 1:** Biochemical features of the patient at baseline and over follow-up.

Measurement	T0 3.5 yrs	T1 3.6 yrs	T2 3.7 yrs	T3 4 years	Reference range
Serum metabolites					
pH	7.25	7,35	7,39	7,36	7.35-7.45
Bicarbonates mmol/L	20.5	24,6	26,7	30,7	21-26
Sodium mEq/L	141	140	138	139	135-145
Chloride mEq/L	110	106	102	103	96-115
Potassium mEq/L	4.2	4,6	4,2	4,3	3,4-5,5
Magnesium mg/dl	2.1	2	2	2,2	1,6-2,6
Phosphate mg/dl	3,2	5,4	5,5	4,8	4,3-5,4
Total calcium mg/dl	9,3	9,2	9,4	8,6	8,6-11
Creatinine mg/dl	0,21	0,26	0,32	0,34	0,20-0,45
Urea mg/dl	14	19	21	20	10-38
Uric acid mg/dl	2.8	4.1	4	3,8	2,5-5
Total protein g/dl	6,6	6,2	6,2	6,4	5,6-7,8
Albumin g/dl	4.1	4,2	4,4	4,6	3,8-5,4
Albumin/globulin ratio	1.6	2,2	2,3	2,6	1,2-2,4
Alkaline phosphatase U/L	1289	694	464	364	140-350
Parathyroid hormone pg/ml	417	223	48,13	82	15-65
25-hydroxy vitamin D ng/ml	6	–	8,84	14	30-100
1,25-dihydroxy vitamin D pg/ml	13,4	–	–	–	20-80
Cystatin C mg/L	–	–	1.01	0,95	0.61-1.1
FGF23 ng/l	7,9	-	–	–	23,2-95,4
Renal tubular screen (urine)					
Urinary pH	8	7.5	–	7	5-7
TRP %	10,7	93,7	–	92,5	85-95
TmP/GFR %	0,34	1,66	–	1,43	2,9-6,5
Urinary uric acid mg/dl	105.9	–	–	75	37-92
Calcium/creatinine mg/mg	–	0.06	–	0.04	0.02-0.2
Urinary sodium mEq/L	363	–	–	155	54-150
Urinary chloride mEq/L	226	–	–	151	46-168
Urinary protein mg/24 h	–	–	115	–	0-150
Urinary calcium mg/24 h	–	–	20.6	–	100-300
Urinary urea mg/24 h	–	–	8304	–	20000-35000
Urinary sodium mEq/24 h	–	–	62	–	40-220
Urinary phosphate mg/24 h	–	–	442.2	–	400-1300
Urinary potassium mEq/24 h	–	–	20.5	–	25-125
Urinary chloride mEq/24 h	–	–	60	–	110-250

## Diagnostic assessment and therapeutic intervention

Investigation of urine metabolites demonstrated low tubular reabsorption of PO4 (TRP) and a reduced tubular maximum reabsorption of PO4 per glomerular filtration rate (TMP/GFR), confirming that renal PO4 loss was the key defect responsible for rickets. Further work-up revealed non-anion gap metabolic acidosis along with urinary loss of bicarbonates, uric acid, amino acids, suggesting renal Fanconi syndrome (**Table 1**). Moreover, FGF23 levels were found to be low (7.9 ng/L, nv), indicating FGF23-independent mechanisms beyond rickets. Subsequently, targeted exome sequencing was requested, with clinical questions being hypophosphatemic rickets and renal Fanconi syndrome.

The child was commenced on Joulie solution (70 mg/kg/day of PO4), calcitriol (0.03 mcg/kg/day), ergocalciferol (1000 IU/day), bicarbonates, and potassium citrate supplementation. Genetic testing revealed a pathogenic heterozygous mutation in the solute carrier family 34 member 1 (SLC34A1) gene (NM_003052.4 C.1006 + 1 G>A splice-site), encoding the renal sodium–inorganic PO4 cotransporter NaPi-IIa, which mediates sodium-dependent PO4 reabsorption in the proximal renal tubule cells.

Surprisingly, within two weeks the biochemical picture normalised, including alkaline phosphatase, PTH, PO4, uric acid, and bicarbonate levels. Consequently, PO4 and bicarbonate supplementation were discontinued, while calcitriol and ergocalciferol supplementation were maintained, alongside potassium citrate to reduce the risk of nephrolithiasis. Despite discontinuation of PO4 supplementation, PO4 concentrations remained stable, and no signs of proximal tubular dysfunction reappeared, underscoring complete and stable reversal of renal Fanconi syndrome (**Table 1**).

**Figure 2:**
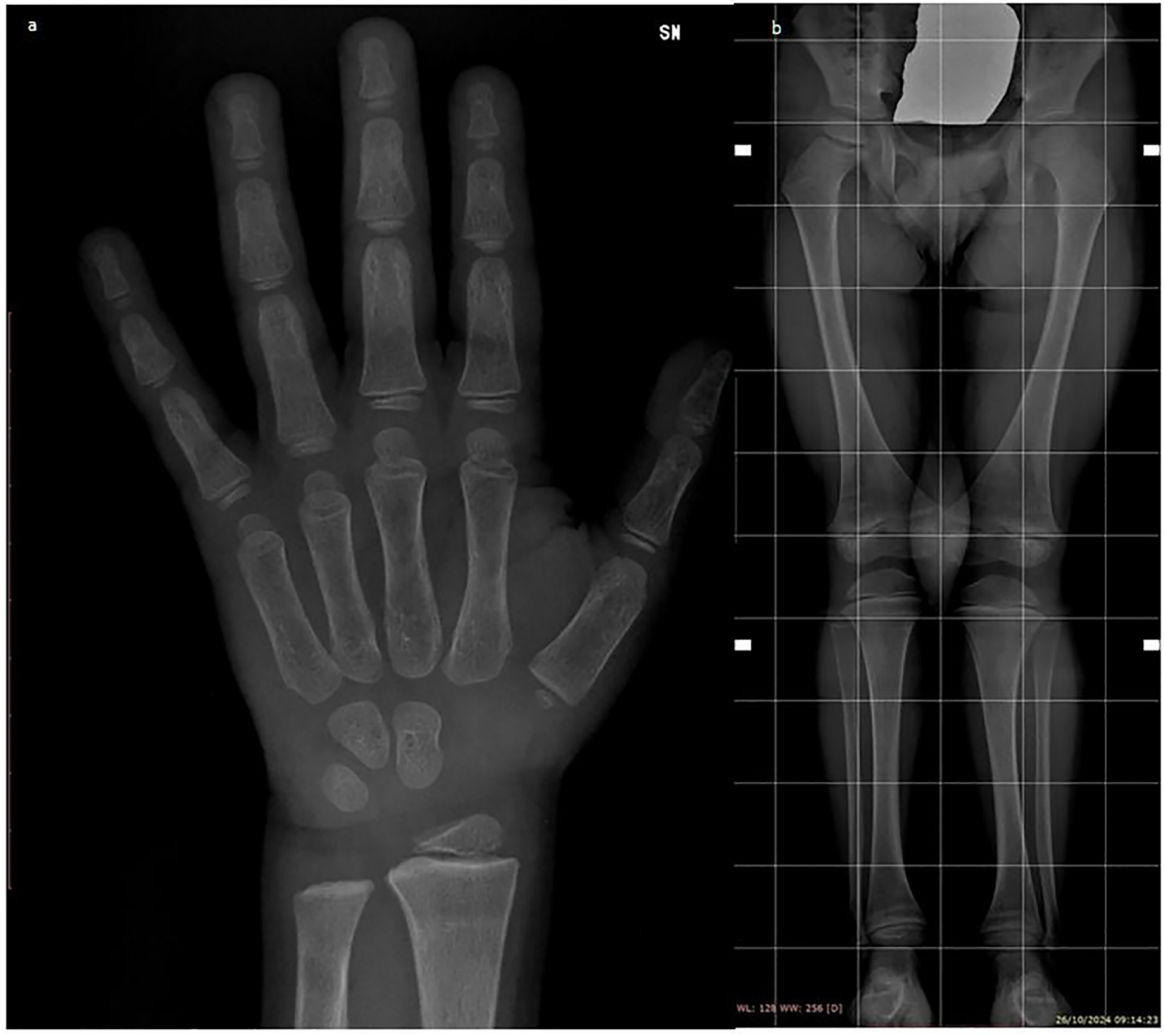
Radiographic improvements in rachitic changes of the wrist **(a)** and knees **(b)** after six months of treatment.

Six months after starting treatment, the genus valgus knee deformity was almost resolved, with a reduction of the intermalleolar distance by approximately 7 cm, and a repeated X-ray revealed marked improvement of rachitic changes at the level of the wrist (**Figure 2a**) and knees (**Figure 2b**). 25(OH)D concentrations were still suboptimal, so that the dose of ergocalciferol was increased to 3000 IU/day, as for age-specific recommendations. Given the absence of epileptic episodes over the previous two years, progressive reduction in the dose of phenobarbital has been planned, possibly associated to weaning of calcitriol.

## Discussion

Rickets associated with renal Fanconi syndrome due to vitamin D deficiency is an extremely rare condition and has never been reported so far in a child taking phenobarbital with no other clear-cut risk factors for vitamin D deficiency. Therefore, our case contributes to raise awareness among the physicians regarding the possible severe renal issues that children at risk to develop vitamin D deficiency may face. Moreover, our patient harboured a pathogenic variant in the *SLC34A1* gene encoding the renal sodium–inorganic PO4 cotransporter NaPi-IIa, thus prompting discussion regarding the pitfalls in the differential diagnosis of rickets in the era of next generation sequencing technologies.

Rickets is characterised by abnormal differentiation and maturation of chondrocytes and deficient mineralisation of the cartilage at the level of growth plate, causing bone deformity (6, 7). It may result from nutritional deficiencies or genetic defects (7).

Calciopenic rickets can be caused by vitamin D or dietary calcium deficiencies, and abnormalities of vitamin D metabolism and action (7). The main risk factors for vitamin D deficiency (8) are summarised in **Table 2**. On the other hand, phosphopenic rickets can be due to inadequate dietary PO4 intake or reduced gastrointestinal PO4 absorption or increased renal losses due to impaired PO4 reabsorption (7).

**Table 2:** Main risk factors for vitamin D deficiency in children [modified from ref. (8)].

Category	Risk factor	Description
Demographic	Age	Infants and young children are at higher risk due to increased demands or reduced skin synthesis.
	Ethnicity	Individuals with darker skin have reduced vitamin D synthesis due to higher melanin levels.
	Geography	Living in regions with low sunlight exposure, high latitudes, or areas with significant air pollution limits ultraviolet-B radiation.
Lifestyle factors	Limited sun exposure	Indoor lifestyle, clothing covering most of the skin, or cultural practices can reduce ultraviolet-B exposure.
	Dietary intake	Inadequate dietary sources of vitamin D, particularly in populations consuming few fortified foods or fatty fish.
Health-related factors	Obesity	Adipose tissue can sequester vitamin D, reducing its bioavailability.
	Malabsorption	Conditions like celiac disease, Crohn’s disease, or cystic fibrosis impair vitamin D absorption.
	Chronic kidney or liver disease	Impaired conversion of vitamin D to its active form.
	Medications	Certain drugs (e.g., enzyme-inducing anticonvulsants, glucocorticoids) can interfere with vitamin D metabolism.
Maternal and infant risk factors	Maternal vitamin D deficiency	Reduced vitamin D delivery to the foetus.
	Exclusive breastfeeding	Breast milk is a poor source of vitamin D, particularly if the mother is deficient.

Of note, in both categories rachitic changes are due to reduced ambient PO4 supply, which impairs caspase-mediated apoptosis of hypertrophic chondrocytes at the level of the growth plate (9). In addition, hypophosphatemia is a frequent finding even in the case of calciopenic rickets, possibly resulting from secondary hyperparathyroidism or other mechanisms (7). In this regard, our case highlights that, in the context of vitamin D deficiency, tubular dysfunction with renal PO4 leak may be the key factor causing or exacerbating hypophosphatemia.

To our knowledge, so far only anecdotal cases of children presenting with vitamin D deficiency-induced proximal renal tubular acidosis or global dysfunction have been reported in the literature (3, 10–19). This condition is characterised by isolated renal tubular acidosis or global reduction in renal reabsorption of PO4, glucose, amino acids, uric acid and bicarbonates, configuring renal Fanconi syndrome (4). When present, symptoms are highly nonspecific, with possible presence of loss of appetite, recurrent vomiting, failure to thrive (10), polyuria and polydipsia (14). Proximal renal tubular acidosis (20) or Fanconi syndrome (14, 21) have also been documented in a few adults with vitamin D deficiency, mainly related to nutritional deficiency or gastrointestinal problems.

The mechanisms behind this condition are complex and only partially unrevealed (3, 10). After entering proximal tubule cells, 25(OH)D is converted by mitochondrial enzyme 1-α-hydroxylase (which in the kidney is only expressed in the proximal nephron site, likely due to the presence of a cell-specific enhancer) into 1,25(OH)_2_D (22, 23). This latter is the active hormone as it can activate along with retinoic acid the heterodimer formed by the vitamin D receptor (VDR) plus the retinoid X receptor (RXR), which translocates into the cell nucleus, where it plays crucial roles in regulating the expression of genes bearing a vitamin D response element (VDRE) involved in renal transporters synthesis and renal function (4, 5). Several studies in humans (24–26) and rodent (27) have provided insights regarding the mechanisms by which vitamin D deficiency can disrupt PO4 and amino acid reabsorption in the proximal nephron site, independent of PTH levels (28), and dietary intake of calcium and PO4 (4, 24–26). Aminoaciduria is also observed in genetic conditions involving vitamin D metabolism and action, such as defects in 1-α-hydroxylase and VDR (3, 4). Instead, the mechanisms behind renal losses of bicarbonates are still largely unknown (4, 29), since the presence of a VDRE in the promoter region of SLC4A4 or SLC9A3 genes, encoding the electrogenic sodium bicarbonate cotransporter of the basolateral membrane NBCe1 and the Na+/H+ exchanger NHE3 at the level of proximal tubule, respectively, remains a hypothesis (30). Based on these considerations, it is conceivable that restoration of circulating concentrations of 1,25(OH)2D in our patient following appropriate supplementation may have reverted proximal renal tubular dysfunction through the up-regulation of transporters synthesis at that level (4).

Regarding the causes of vitamin D deficiency in our patient, even though poor nutritional intake could not be reliably ruled out, the most likely determinant was long-term phenobarbital treatment, which is known to decrease vitamin D bioavailability (7, 31–33).

Specifically, phenobarbital suppresses the expression of two critical enzymes catalysing the 25-hydroxylation step, namely CYP27A1 and CYP2D25, both at the transcriptional level, through pregnane X receptor (PXR)-mediated downregulation of the CYP2D25 promoter in hepatic cells, and/or by reducing CYP2D25 mRNA stability (32). Moreover, phenobarbital accelerates the catabolism of 25(OH)D and 1,25(OH)_2_D to inactive metabolites, mainly by inducing the activity of the CYP3A4 enzyme in the liver (32). It is worth noting that in a previous case described by Taylor et al. (14) revolving around a 33-year-old African American woman with renal Fanconi syndrome and altered bone metabolism taking long-term phenytoin treatment for epilepsy, this was not listed among the possible factors believed to contribute to vitamin D deficiency. This looks surprising as phenytoin may reduce vitamin D bioavailability with mechanisms similar to phenobarbital (34). Indeed, 10 to 30 percent of adult patients taking phenobarbital or phenytoin exhibit radiological or biochemical evidence of vitamin D deficiency (35). Hence, ongoing debate exists as to whether children taking anti-epilepsy medications should receive routine vitamin D supplementation. In this respect, our case confirms the need to implement such a strategy at least in children taking enzyme-inducing drugs, in order to prevent nutritional rickets and its severe health consequences. This is in agreement with the Global Consensus recommendations for prevention and management of nutritional rickets made by a panel of experts in 2016, which strongly recommended vitamin D supplementation in children beyond 1 year of age at high risk of vitamin D deficiency, including those who have conditions that reduce synthesis or intake of vitamin D (36). The American Academy of Pediatrics recommends 400 to 1000 IU whereas the Endocrine Society and the Global consensus suggest 600 to 1000 IU and at least 600 IU daily, respectively, for children who are at an increased risk to develop vitamin D deficiency (8, 37). However, it is still unclear whether subjects affected with conditions that may alter vitamin D metabolism require higher prophylactic doses, compared to other risk categories (8, 37).

A further issue warranting comment is the presence of the pathogenic variant in the SLC34A1 gene encoding the renal sodium–inorganic PO4 cotransporter NaPi-IIa. This latter is an ATP-powered transporter located in the apical membrane of proximal renal tubule cells, mediating sodium-dependent PO4 reabsorption (38). Mice lacking NaPi-IIa exhibit hypophosphatemia and hyperphosphaturia (39).

Homozygous loss-of-function mutations of the SLC34A1 gene have been reported to cause vitamin D-resistant hypophosphatemic rickets, associated with renal Fanconi syndrome, elevated serum 1,25(OH)_2_D (stimulated by hypophosphatemia), and subsequent hypercalciuria (40, 41) due to enhanced intestinal calcium absorption (42, 43). From a functional point of view, expression studies in cellular models revealed a failure of the mutant transporter to reach the plasma membrane (40).

Several heterozygous variants in SCL34A1 have been hypothesised to cause renal PO4 losses; however functional characterisation of these variants revealed negligible effects on PO4 homeostasis (44–46). More recently, heterozygous SLC34A1 mutations have been associated with development of kidney stones (47) or hypophosphatemic kidney stones with osteoporosis (48), even if often in the absence of functional verification. Although based on the current state of knowledge our case could be considered a healthy carrier of monoallelic pathogenic variants in SLC34A1 (46–44), given the extremely uncommon occurrence of renal tubular involvement in the context of severe vitamin deficiency, we speculate that the presence of the genetic variant might represent a key factor triggering or accelerating such an association.

In our case, the complete and persistent reversal of urine and serum abnormalities upon starting vitamin D replacement helped direct the diagnosis towards vitamin deficiency, rather than FGF-23 independent inherited tubulopathy. Indeed, in the latter case PO4 concentrations invariably do not rise up to the normal reference range for age, despite receiving huge and frequent PO4 substitution (49). Moreover, serum alkaline phosphatase in our patient was markedly raised, primarily due to increased osteoclastic activity resulting from vitamin D deficiency and compensatory hyperparathyroidism, while only modest increases are often seen in genetic forms of phosphopenic rickets (7). Finally, the relatively late onset of rachitic changes in our patient also made a diagnosis of inherited tubulopathy unlikely, as this usually presents already in infancy, or when the patient acquires the upright position (50).

## Conclusion

In conclusion, our case provides further evidence that severe vitamin D deficiency can induce global proximal renal tubular dysfunction leading to metabolic derangement and hypophosphatemic rickets. Although the prevalence of nutritional rickets has largely decreased over the last decades in industrialised countries, due to the widespread diffusion of vitamin D prophylaxis, it remains a significant health issue especially in subjects with risk factors, such as administration of enzyme-inducing anticonvulsants. Indeed, the current case report emphasises the critical importance of routine vitamin D supplementation in these categories, in order to prevent severe health consequences.

Finally, this case highlights the pitfalls in the differential diagnoses between calciopenic and phosphopenic rickets, as well as between nutritional and inheritable causes of rickets. Whether monoallelic variants of the SLC34A1 may favour the development of proximal renal tubular dysfunction in subjects with vitamin D deficiency needs to be evaluated in further studies.

## Data Availability

The raw data supporting the conclusions of this article will be made available by the authors, without undue reservation.
